# Cobalt(II)-coordination polymers containing glutarates and bipyridyl ligands and their antifungal potential

**DOI:** 10.1038/s41598-019-50258-1

**Published:** 2019-10-18

**Authors:** Hyun-Chul Kim, Sarmistha Mitra, Mayura Veerana, Jun-Sup Lim, Hye-Ryeon Jeong, Gyungsoon Park, Seong Huh, Sung-Jin Kim, Youngmee Kim

**Affiliations:** 1 Department of Chemistry and Protein Research Centre for Bio-Industry, Hankuk University of Foreign Studies, Yongin, 17035 Korea; 2 Plasma Bioscience Research Center and Department of Electrical and Biological Physics, Kwangwoon University, Seoul, 01897 Republic of Korea; 3 Institute of Nano-Bio Technology and Department of Chemistry and Nano Science, Ewha Womans University, Seoul, 120-750 Korea

**Keywords:** Chemistry, Materials science

## Abstract

Three new Co^II^-coordination polymers (Co-CPs) containing glutarates and bipyridyl ligands, formulated as [Co_2_(Glu)_2_(µ-bpa)_2_]·(H_2_O)_4_ (**1**), [Co_4_(Glu)_4_(µ-bpp)_2_] (**2**), and [Co_2_(Glu)_2_(µ-bpe)_2_]·(H_2_O)_0.5_ (**3**), were prepared, and their structures were determined by X-ray crystallography. Glutarates bridge Co^II^ ions to form 2D sheets, and the sheets are connected either by bpa or by bpp ligands to form 3D networks **1** and **2**, respectively. Both frameworks **1** and **2** are two-fold interpenetrated, and there is no significant void volume in either network. Four glutarates bridge two Co^II^ ions to form chains, and these chains are connected by bpe ligands to form the 2D sheet **3**. The antifungal properties of these new Co-CPs were tested against two model fungal pathogens, *Candida albicans* and *Aspergillus niger*. Under the maximum concentration of Co-CPs, 2.0 mg mL^−1^, the inhibition rates of Co-CPs against *A. niger* were much lower (44–62%) than those (90–99.98%) observed in *C. albicans*. The results indicate that **1**–**3** can inactivate *C. albicans* cells more efficiently than *A. niger* spores in the same treatment time, and the greater inactivation of *C. albicans* can be explained by dramatic changes in the morphology of *C. albicans* cells. We also found that Co-CPs could generate the reactive species NO and H_2_O_2_, and these species might play a role in inactivating fungal cells. Additionally, degradation tests confirmed that the leaching of Co^II^ ions from Co-CPs was not significant. The small amount of leached Co^II^ ions and the robust Co-CPs themselves as well as the reactive species generated by Co-CPs can actively participate in fungal inactivation.

## Introduction

Metal-organic frameworks (MOFs) containing large void volumes after the removal of captured solvent molecules^1–4^ can be valuable for a wide range of applications, including separation^5–7^, CO_2_ capture^8–10^, H_2_ storage^11–13^, heterogeneous catalysis^14–16^, and biomedical usage^17,18^. The properties of MOFs can depend on many factors, such as the metal ion, rigidity/flexibility, chemical functionality, and geometry of the framework. MOFs can be tuned by changing bridging ligands. The flexible α,ω–alkane (or alkene) dicarboxylates have been utilized as bridging ligands by our group and others for constructing new functional MOFs^19–21^. A systematic study of MOFs containing these α,ω-alkane (or alkene)-dicarboxylate ligands has broadened the scope of MOF materials.

Glutarate is a flexible dicarboxylate ligand that can bridge Cu^II^ ions to form two-dimensional (2D) sheets, and bipyridyl ligands can be used to connect these sheets to form three-dimensional (3D) frameworks, Cu-MOFs^19,22–26^. We and other groups have studied Cu-MOFs containing these glutarates and bipyridyl ligands. Our group has reported 3D Cu-MOFs containing glutarates and the bipyridyl ligands (bpa = 1,2-bis(4-pyridyl)ethane and bpp = 1,3-bis(4-pyridyl)propane). Both MOFs contain Cu_2_ paddle-wheel units connected by glutarates to form 2D sheets, and the sheets were also bridged by either bpa or bpp to form 3D frameworks^19^. They showed good selectivity for CO_2_ over N_2_ and H_2_. Esterhuysen, Barbour and coworkers performed a systematic study of three closely related microporous Cu-MOFs containing glutarates and bipyridyl linkers, namely, bpy, bpe, and bpymh (bpy = 4,4′-bipyridine, bpe = 1,2-bis(4-pyridyl)ethylene, bpymh = N,N′-bis(pyridine-4-ylmethylene)hydrazine)^22^. These linkers increased the pore dimensions for the gas sorption behaviour of the frameworks. Solvent- and pressure-induced phase changes in the same 3D Cu-MOFs *via* glutarate conformational isomerism have also been reported^23^. Recently, these water-stable robust Cu-MOFs containing glutarates and bipyridyl linkers (bpy, bpa, bpe, and bpp) were tested for antibacterial activities against five bacteria^27^. They exhibited excellent antibacterial activities against five kinds of bacteria tested, including both Gram-positive bacteria (*Staphylococcus aureus* and *MRSA*) and Gram-negative bacteria (*Escherichia coli*, *Klebsiella pneumonia*, and *Pseudomonas aeruginosa*), with very low MBCs (minimal bactericidal concentration).

**Scheme 1:**
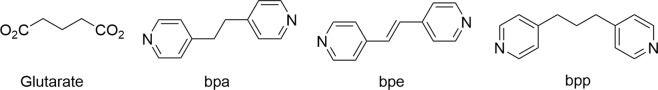
The chemical structures of glutarate, bpa, bpp, and bpe ligands.

As an extension of our previous work, we present here two new 3D Co^II^-coordination polymers (Co-CPs) containing glutarates and bipyridyl ligands, formulated as [Co_2_(Glu)_2_(µ-bpa)_2_]·(H_2_O)_4_ (**1**) and [Co_4_(Glu)_4_(µ-bpp)_2_] (**2**) (Glu = glutarate) for a systematic study of MOFs and CPs containing glutarates and bipyrdyl ligands. We also present a new 2D Co-CP containing bipyridyl ligands with double bonds, formulated as [Co_2_(Glu)_2_(µ-bpe)_2_]·(H_2_O)_0.5_ (**3**). They were prepared by a solvothermal reaction in a DMF solution of the mixture of Co(NO_3_)_2_, glutaric acid and bpa, bpp, or bpe (Scheme 1). The antifungal properties of these new Co-CPs were tested against two model fungal pathogens, *Candida albicans* and *Aspergillus niger*. *C. albicans* is an opportunistic fungal pathogen that can infect human tissues, causing candidiasis^28^. *C. albicans* occurs mostly in yeast form but becomes filamentous during infection^29^. *A. niger* is a filamentous Ascomycete fungus implicated in human and plant infection as well as food contamination and fermentation^29^. This fungus is often found in bakery products, intermediate-moisture food products, cheeses, preserved fruits and grains. Although it has been reported that the water-stable Cu-BTC MOF (BTC = 1,3,5-benzenetricarboxylate) can inhibit the growth rate of *C. albicans*^30^, and other Cu-based HKUST-1 MOF can inactivate *Saccharomyces cerevisiae* and its antifungal property is due to releasing Cu^II^ ions from its own structure^31^, Co-based MOFs and CPs have rarely been tested for their antifungal properties. A Co-based MOF (Co-TDM, TDM^8−^ = [(3,5-dicarboxyphenyl)-oxamethyl]methane) was shown to be highly effective at inactivating *E. coli*^32^. However, there are few reports on the antifungal activity of Co-based MOFs and CPs.

## Results and Discussion

### Structure description

**Figure 1:**
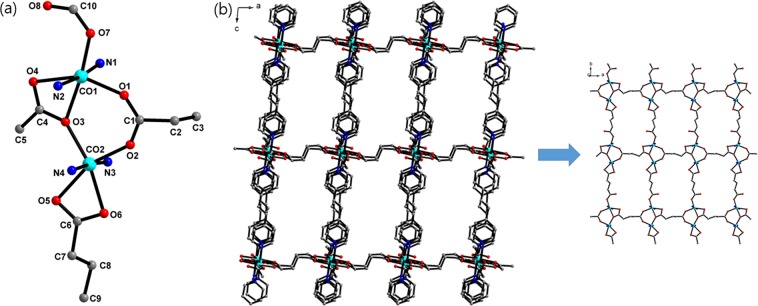
(**a**) Coordination environment around Co^II^ centres of **1**. (**b**) Crystal structure of **1** along the *b* axis with a 2D sheet. All hydrogens and disordered atoms are omitted for clarity. The colour codes: green, Co; red, oxygen; blue, nitrogen; grey, carbon.

**Figure 2:**
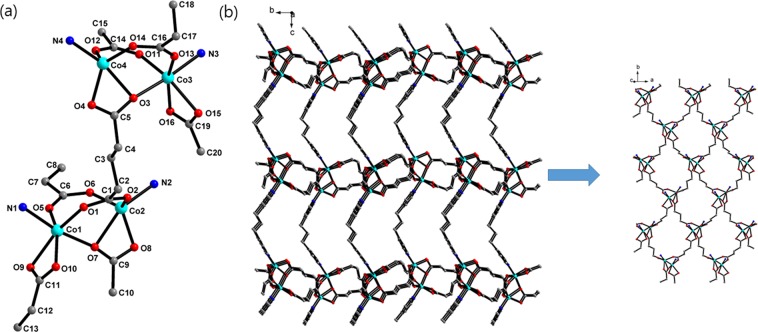
(**a**) Coordination environment around Co^II^ centres of **2**. (**b**) Crystal structure of **2** along the *a* axis with a 2D sheet. All hydrogen atoms are omitted for clarity. The colour codes: green, Co; red, oxygen; blue, nitrogen; grey, carbon.

Co-CPs **1** and **2** crystallize in the monoclinic *P2*_1_*/n* space group, and single-crystal X-ray analysis (Table 1) revealed that the glutarates bridge Co^II^ ions to form two-dimensional (2D) sheets, and the sheets are also connected either by bpa ligands or by bpp ligands to form 3D networks **1** (Fig. 1) and **2** (Fig. 2), respectively. In **1**, two glutarates bridge two Co^II^ ions in bridging mode and chelating/bridging mode to form one-dimensional (1D) chains, and these chains are connected by other glutarates in chelating and monodentate coordination modes to form a 2D sheet. The bpa ligands connect these 2D sheets to form a 3D network. The 3D networks are two-fold interpenetrated (Fig. S1a), and there is no significant void volume for solvents (4.9%, 161.5 Å^3^/3297.9 Å^3^ on PLATON analysis^33^). The coordination geometry of one Co^II^ ion is distorted octahedral constructed by one bridging carboxylate O atom (η^1^:η^1^:μ_2_), two chelating O atoms (η^1^:η^1^), one monodentate carboxylate O atom (η^1^:η^0^), and two pyridyl N atoms. That of the other Co^II^ ions is also distorted octahedral constructed by one bridging O atom (η^1^:η^1^:μ_2_), two chelating O atoms (η^1^:η^1^), one chelating/bridging O atom (η^2^:η^1^:μ_2_), and two pyridyl N atoms. The Co-O bond distances range from 1.994(4) to 2.285(4) Å, and the Co-N distances range from 2.150(3) to 2.184(3) Å (Table 2). The Co···Co distance in a Co_2_ unit is 3.804 Å.

**Table 1:** Crystallographic Data for 1–3.

	1	2	3
Empirical formula	C_34_H_13_Co_2_N_4_O_8_	C_46_H_52_Co_4_N_4_O_16_	C_34_H_32_Co_2_N_4_O_8_
Formula weight	723.34	1152.63	742.49
Temperature (K)	296(2)	296(2)	296(2)
Wavelength (Å)	0.71073 Å	0.71073 Å	0.71073 Å
Space group	P 2_1_/n	P 2_1_/n	P -1
a (Å)	9.1670(3)	10.5398(3)	10.5529(3)
b (Å)	27.1849(8)	28.1874(7)	13.0494(3)
c (Å)	13.5929(4)	16.7751(4)	13.7009(3)
α (°)	90.00	90.00	69.9765(15)
β (°)	95.6332(16)	106.4395(12)	67.3583(14)
γ (°)	90.00	90.00	80.2724(16)
Volume (Å^3^)	3371.05(18)	4780.0(2)	1634.57(7)
Z	4	4	2
Density (calc.) (mg/m^3^)	1.425	1.602	1.509
Absorption coeff. (mm^−1^)	1.039	1.439	1.073
Crystal size (mm)	0.08 × 0.14 × 0.34	0.05 × 0.10 × 0.18	0.02 × 0.05 × 0.14
Reflections collected	91224	86389	56080
Independent reflections	5828 [R(int) = 0.0387]	11918 [R(int) = 0.0755]	8111 [R(int) = 0.0952]
Data/restraints/parameters	5828/120/529	11918/0/631	8111/0/433
Goodness-of-fit on F^2^	1.061	1.000	1.014
Final R indices [I > 2σ(I)]	R_1_ = 0.0757, wR_2_ = 0.1903	R_1_ = 0.0516, wR_2_ = 0.1139	R_1_ = 0.0465, wR_2_ = 0.0896
R indices (all data)	R_1_ = 0.0829, wR_2_ = 0.1972	R_1_ = 0.0925, wR_2_ = 0.1277	R_1_ = 0.1049, wR_2_ = 0.1078
Largest diff. peak and hole (e.Å^−3^)	1.542 and −1.442	0.910 and −0.479	0.359 and −0.315
CCDC number	1561941	1561902	1561903

**Table 2:** Selected bond distances for **1**–**3**.

	1	2	3
Co-O	1.994(4)–2.285(4) Å	1.952(3)–2.239(3) Å	2.029(2)–2.260(2) Å
Co-N	2.150(3)–2.184(3) Å	2.048(3)–2.154(3) Å	2.153(2)–2.168(2) Å
Co∙∙∙Co	3.799 Å	3.49, 3.52 Å	4.011, 4.018 Å

For **2**, two Co^II^ ions are bridged by three glutarates in two bridging and one chelating/bridging modes, and one of the Co^II^ ions is chelated by the other glutarate to form 2D sheets. These 2D sheets are connected by bpp ligands to form a 3D network. The 3D networks are also two-fold interpenetrated (Fig. S1b), and there is no void volume for solvents. The coordination geometry of one Co^II^ ion is distorted square pyramidal was constructed by two bridging O atoms, two chelating O atoms, and one pyridyl N atom and that of the other Co^II^ ion is distorted octahedral constructed by two bridging O atoms, two chelating O atoms, one chelating/bridging O atom, and one pyridyl N atom. The Co-O bond distances range from 1.952(3) to 2.239(3) Å, and the Co-N distances range from 2.048(3) to 2.154(3) Å (Table 2). The Co···Co distances in Co_2_ units are 3.49 and 3.49 Å.

The 3D networks of both **1** and **2** indicate a 6-connected uninodal net (**pcu** alpha-Po primitive cubic) with a Schläfli symbol of 4^12^·6^3^, assuming that the Co_2_ unit acts as a node by ToposPro analysis^34^.

**Figure 3:**
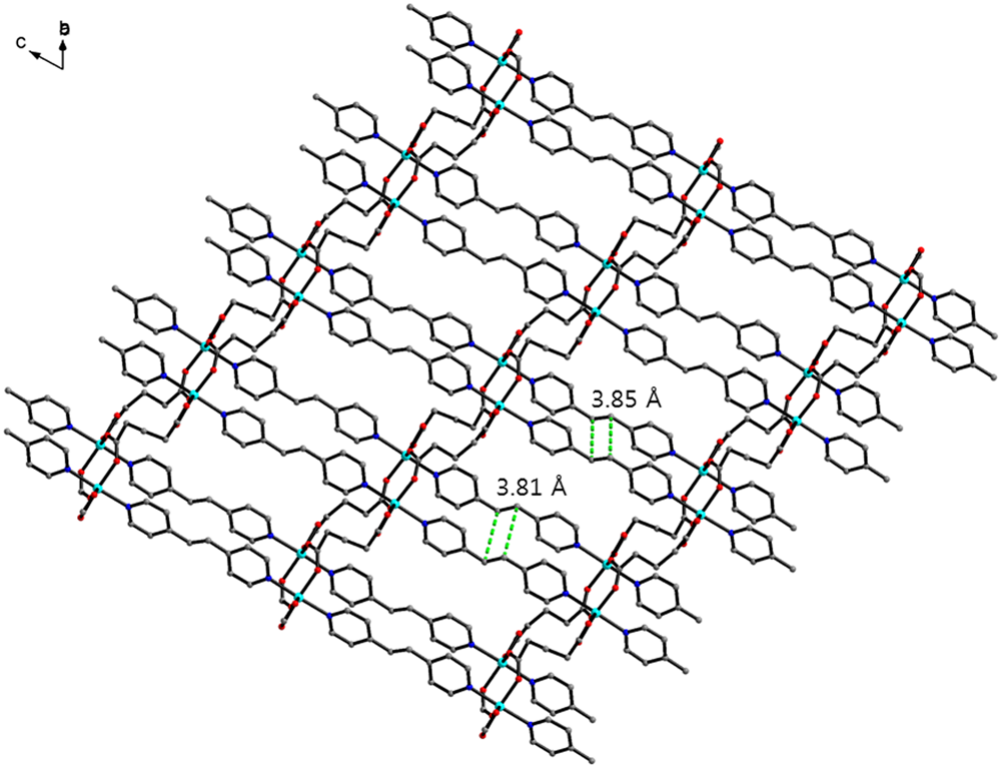
The 2D structure of **3**. The green dotted lines are the distances between C=C double bonds. All hydrogens and disordered atoms are omitted for clarity. The colour codes: green, Co; red, oxygen; blue, nitrogen; grey, carbon.

Co-CP **3** crystallizes in a triclinic space group, and X-ray analysis (Table 1) shows that four glutarates bridge two Co^II^ ions to form chains, and these chains are connected by bpe ligands to form a 2D sheet (Fig. 3). The coordination geometry of a Co^II^ ion is distorted octahedral constructed by two bridging O atoms from the different glutarates, two chelating O atoms from glutarate, and two pyridyl N atoms. The C=C double bonds of the bpe ligands are close enough to produce photoreaction [2 + 2] addition products, but no photoaddition reaction occurred in Co-CP **3**. The distances between double bonds are 3.81 and 3.85 Å (Fig. 3). The Co-O bond distances range from 2.029(2) to 2.260(2) Å, and the Co-N distances range from 2.153(2) to 2.168(2) Å (Table 2). The Co···Co distances in Co_2_ units are 4.011 and 4.018 Å.

**Scheme 2:**
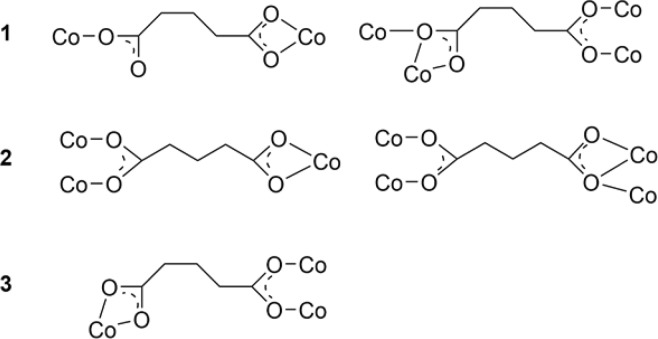
Coordination modes and conformations of glutarates in networks **1**–**3**.

The coordination modes of two the carboxylates in a glutarate are shown in Scheme 2. For 3D networks, two types of coordination modes are used: (η^1^:η^0^)(η^1^:η^1^) and (η^2^:η^1^:μ_2_)(η^1^:η^1^:μ_2_) for **1**, (η^1^:η^1^:μ_2_)(η^1^:η^1^) and (η^1^:η^1^:μ_2_)(η^2^:η^1^:μ_2_) for **2**. For 2D sheets, one coordination mode (η^1^:η^1^)(η^1^:η^1^:μ_2_) is used for **3**. The flexible glutarate can have different conformations in coordination compounds^23^, **1** and **3** have a *gauche-trans* conformation, and **2** has a *trans-trans* conformation.

The bulk purities of **1**–**3** were confirmed by powder X-ray diffraction (PXRD) (Figs S2–S4), elemental analysis (see experimental section), and thermogravimetric analysis (TGA) of the as-prepared samples (Figs S5–S7). The scanning electron microscopy (SEM) images of three Co-CPs are shown in Fig. S8. **1** and **3** have rod type crystals, **2** has aggregated crystals. The crystals of all three compounds were used as prepared without grinding. The structural stabilities of **1**–**3** in water or 1x PBS (Phosphate Buffered Saline) solution have been tested by PXRD, and the PXRD patterns of **1** and **2** indicate that their structures are stable after 4 days in water or PBS solution (Fig. S9). For **3** after 4 days in water or PBS, the intensities of diffraction planes of (011), (101), (112), and (1-1-1) at 2θ = 8.63, 9.54, 13.677, and 14.484°, respectively, were reduced compared with those of the as-prepared sample. Contrarily, the diffraction planes of (110) and (1–10) at 2θ = 11.457 and 11.962° were maintained. These differences of diffraction patterns between the as-prepared sample and samples after 4 days in water or PBS imply that partial degradation of the framework. This also consists with the small amount of Co^II^ ions released from Co-CPs in PBS which will be discussed in the later part.

### Antifungal activities

**Figure 4:**
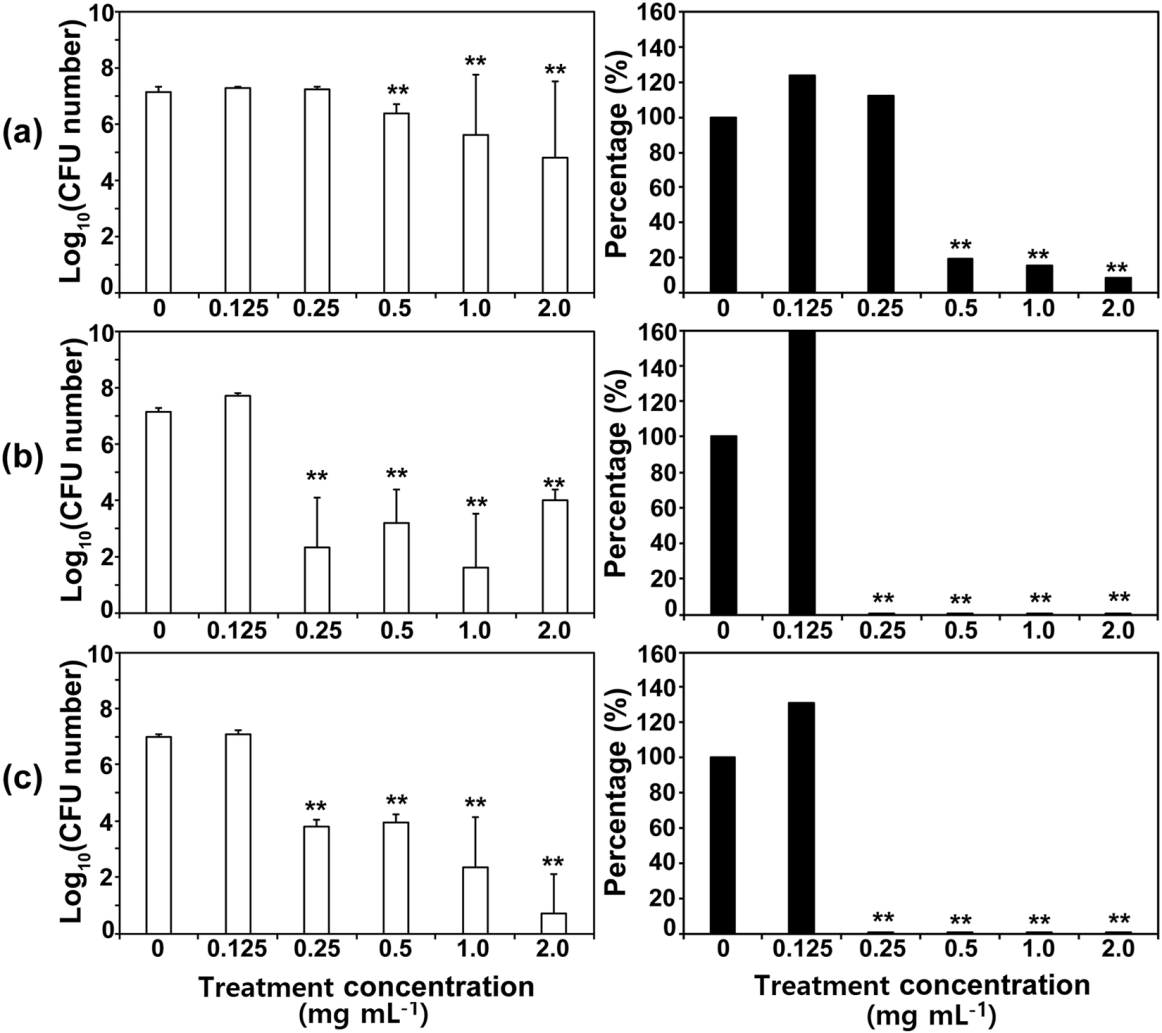
Antifungal activities of **1** (**a**), **2** (**b**), and **3** (**c**) against *Candida albicans* at different concentrations. Graphs on the left show log scale CFU (colony forming unit) numbers of *C. albicans* in each treatment. The percentage in the right graphs was calculated as follows: (CFU number in each compound treatment/CFU number of control) × 100. Each bar represents the average and standard deviation of 9 replicates. Student’s *t*-test was performed between the control and each treatment; **p < 0.01.

**Figure 5:**
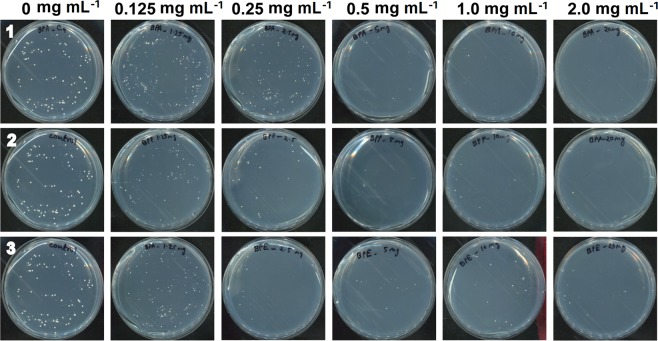
Images of *C. albicans* colonies on PDA plates. After treatment with different concentrations of compounds for 4 days, *C. albicans* cells diluted 10^5^ times were plated on PDA.

The antifungal properties of the three Co-CPs against *C. albicans* and *A. niger* were assessed by using the plate counting method. *C. albicans* cells were incubated in different concentrations of each compound (2, 1, 0.5, 0.25, and 0.125 mg mL^−1^) for 4 days. When yeasts were incubated with compounds for 1 day, no significant antifungal effects were observed (Fig. S10a). However, after 4 days, the difference in CFU numbers between the control and treatments became more dramatic (Figs 4 and S11). Representative images of *C. albicans* colonies on PDA (Potato Dextrose Agar) plates are shown in Fig. 5. **1** inactivated *C. albicans* by approximately 91% at 2 mg mL^−1^, **2** by 99.98% at 0.25 mg mL^−1^, and **3** by 99.93% at 0.25 mg mL^−1^ (Figs 4 and S11). **2** and **3** showed relatively higher antifungal activity against *C. albicans* than **1** (Figs 4 and 5). The result indicates that the viability of yeast cells is reduced by increasing the concentration of Co-CPs, although a relatively longer incubation time is required to obtain a similar level of antifungal effect to that of the previously reported Cu-BTC, in which *C. albicans* was effectively inactivated with a 500 ppm (0.5 mg mL^−1^) concentration and a short incubation time (60 min)^30^.

**Figure 6:**
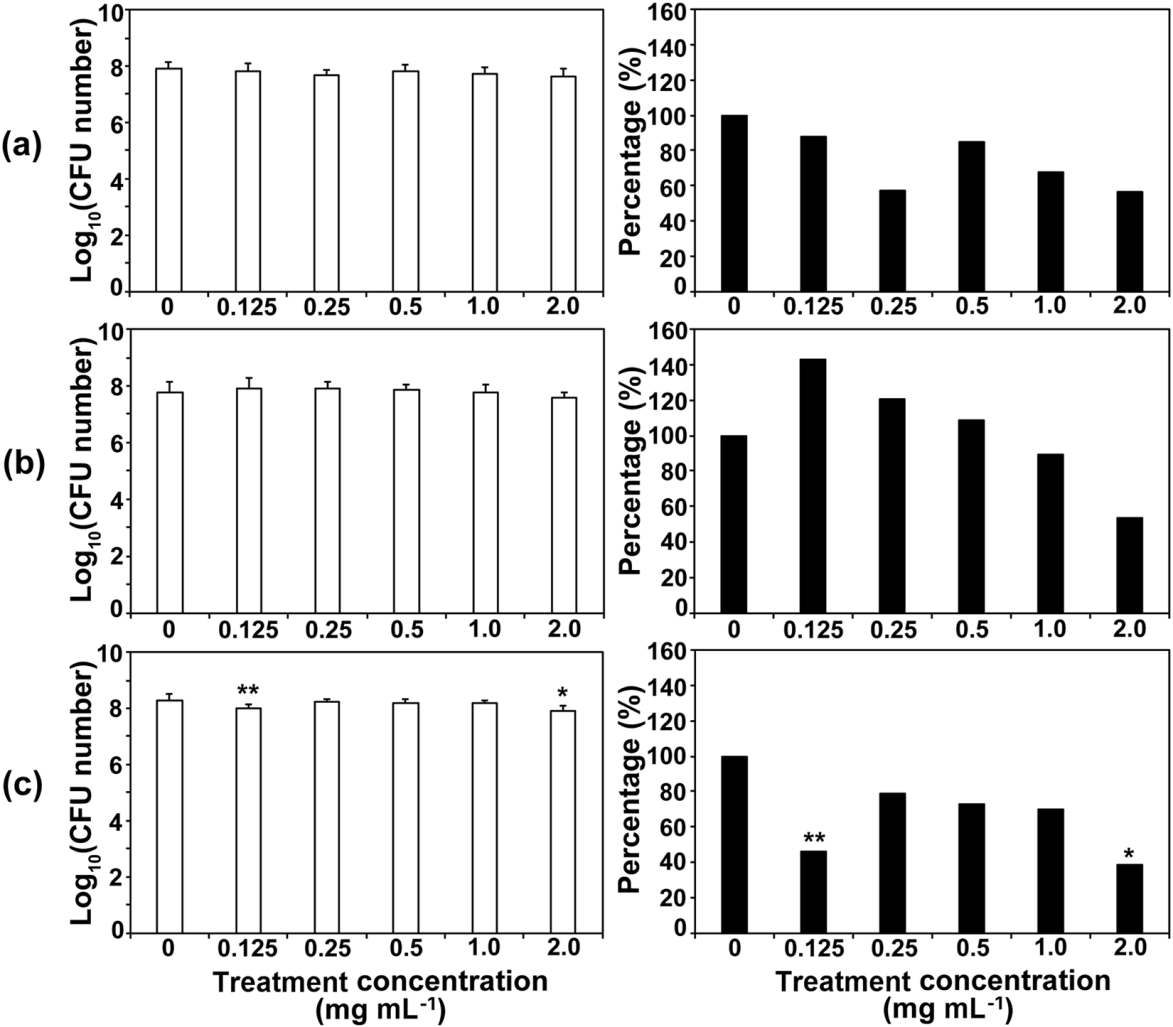
Antifungal activities of 1 (**a**), 2 (**b**), and 3 (**c**) against *Aspergillus niger* at different concentrations. Graphs on the left show log scale CFU (colony forming unit) numbers of *A. niger* in each treatment. The percentage in the right graphs was calculated as follows: (CFU number in each compound treatment/CFU number of control) × 100. Each bar represents the average and standard deviation of 9 replicates. Student’s *t*-test was performed between the control and each treatment; *p < 0.05, **p < 0.01.

**Figure 7:**
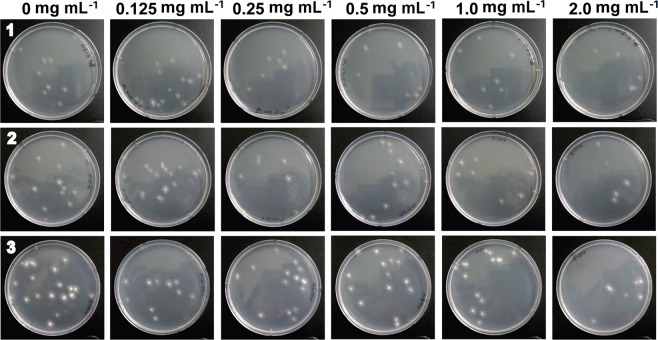
Images of *A. niger* germinated spores on PDA plates. After treatment with different concentrations of compounds for 4 days, *A. niger* spores diluted 10^7^ times were plated on PDA.

*A. niger* spores were also incubated in different concentrations of each compound (2, 1, 0.5, 0.25, and 0.125 mg mL^−1^) for 4 days, and spore germination was assessed as an indicator of spore viability. As observed in *C. albicans*, treatment of *A. niger* spores with compounds for 1 day did not produce any significant antifungal effects (Fig. S10b). In contrast to *C. albicans* cells, *A. niger* spores are pigmented, and this pigmentation may affect the responses to Co-CPs. Inactivation of *A. niger* spores by Co-CPs measured as number and percentage of spore germination was not dramatic compared to that observed in *C. albicans* (Figs 6 and S12). Representative images of *A. niger* colonies on PDA plates are shown in Fig. 7. The germination percentage of *A. niger* spores was more slowly reduced over increased concentrations of compounds compared to the CFU percentage of *C. albicans* cells (Figs 4 and 6 and S11, S12). At the maximum concentration of compounds, 2.0 mg mL^−1^, the inhibitory effect of **1**, **2**, and **3** against *A. niger* were approximately 44%, 46%, and 62%, respectively, after incubation for 4 days (Fig. 6). These inhibition rates were much lower than those (90–99.98%) observed in *C. albicans* (Figs 4 and 6).

Our results indicate that Co-CPs can inactivate *C. albicans* cells more efficiently than *A. niger* spores in the same treatment time. The antifungal activity of Co-CPs is likely to be variable depending on fungal species. The differential sensitivity of fungal species to MOFs was also observed in a previous study in which Cu-BTC inhibited spores of *F. oxysporum* and *A. oryzae* up to 30% but was not affected by *A. niger* spores even after 7 days of incubation^30^. Our results can be compared with those of silver(I) CPs and MOFs showing a notable antimicrobial efficiency against a panel of bacteria (*E. coli, P. Aeruginosa and S. aureus*) and yeast (*C. albicans*) with the minimum inhibitory concentrations (MIC) of 30–60 μg mL^−1^ (normalized MIC of 11–68 nmol mL^−1^), but their antimicrobial action is due to release Ag^I^ ions from Ag-MOFs or Ag-CPs^35–38^. The antifungal activities of Co-CPs can also be compared with those of other nanomaterials: Ag nanoparticles on *C. albicans* biofilms^39^, ZnO nanoparticles against *E. coli*^40^, and reduced graphene oxide nanosheets against *A. niger*, *A. oryzae*, and *F. oxysporum* (*Fusarium oxysporum*) with half-maximal inhibitory concentrations (IC_50_) of 50, 100, and 100 μg mL^−1^, respectively^41^.

**Figure 8:**
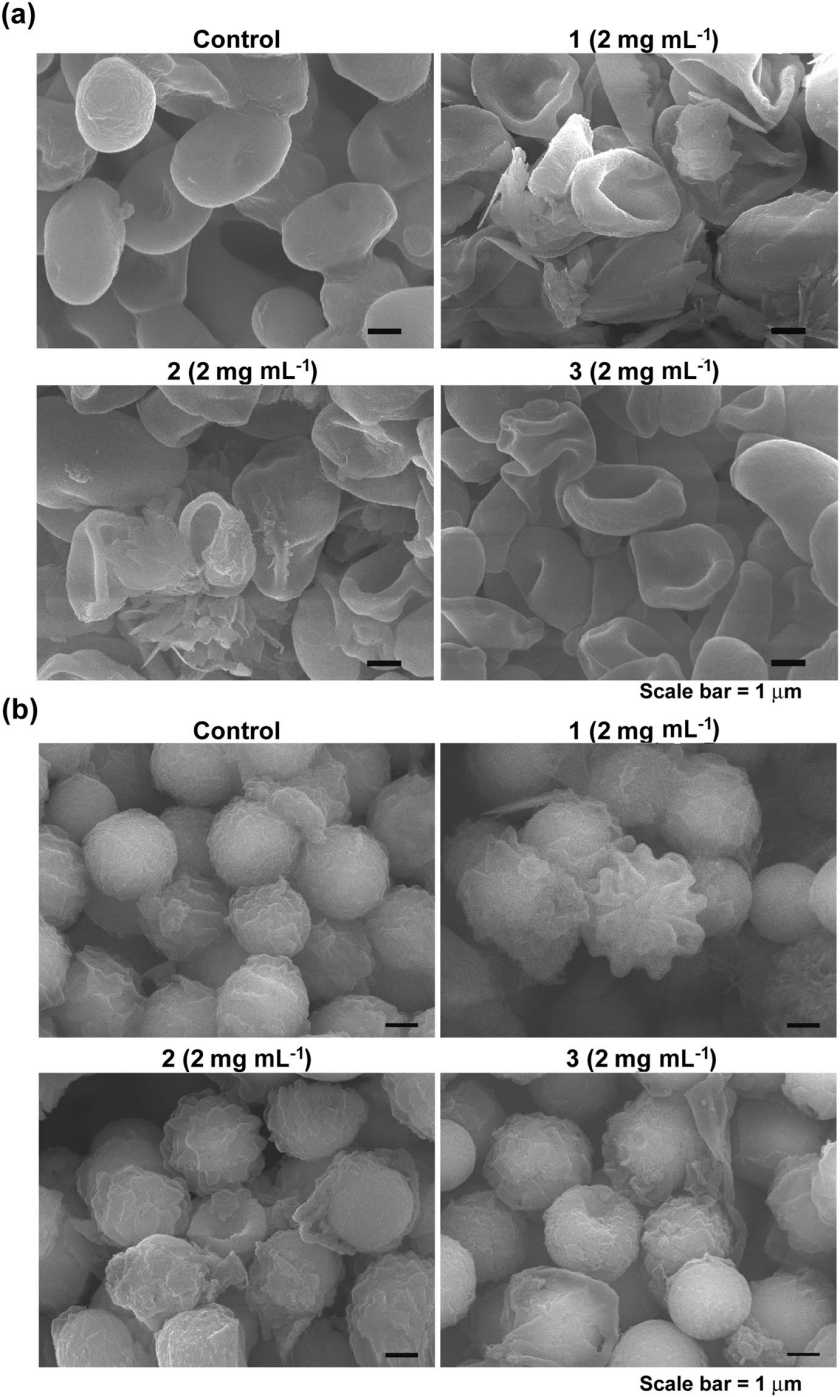
Morphology of *C. albicans* cells (**a**) and *A. niger* spores (**b**) after treatment with Co-CPs.

Our SEM analysis demonstrated that the morphology of *C. albicans* cells and *A. niger* spores was differentially changed after treatment with Co-CPs (Fig. 8). Loosened spore surfaces (probably destruction of the fungal cell wall) without severe changes in cell shape were observed in many *A. niger* spores, while a large proportion of *C. albicans* cells were severely crushed (Fig. 8). In *A. niger* treated with Co-CPs, the spore surface was wrinkled, and the surface layers peeled off (Fig. 8b). This result indicates damage to the spore cell wall, most likely making the spores more vulnerable to mechanical and other environmental stresses. However, we could not see wrinkles or peel-off of the surface in *C. albicans* cells treated with Co-CPs. This may be because of the difference in the composition and structure of the cell wall between *C. albicans* and *A. niger*, although both are fungi. More dramatic changes in morphology were observed in *C. albicans* than in *A. niger*, which might explain the greater inactivation by Co-CPs in *C. albicans*. The morphological changes observed after treatment with Co-CPs were not significantly different among the 3 types of Co-CPs (Fig. 8), where **1** and **2** are two-fold-interpenetrated 3D frameworks and **3** is a 2D sheet.

**Figure 9:**
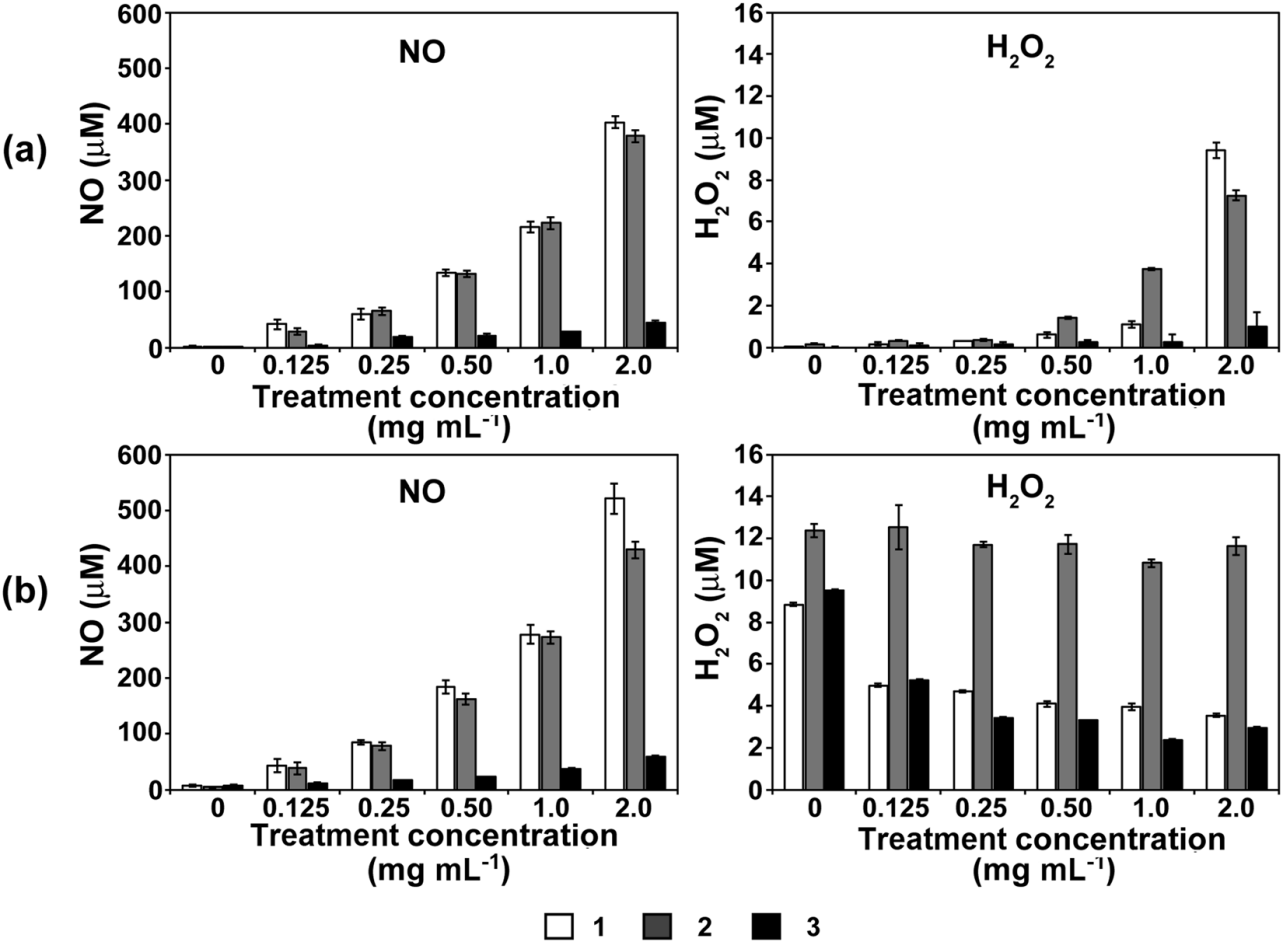
Concentrations of NO (nitric oxide) and H_2_O_2_ in 1 mL of 1x PBS solution after treatment with Co-CPs for 0 day (**a**) and 4 days (**b**).

To find the mechanism for antifungal effects, ROS (reactive oxygen species) and RNS (reactive nitrogen species) levels in PBS solution were analysed after treatment with Co-CPs. These Co-CPs can generate ROS/RNS that can interact with biomolecules in fungal spores, producing an inhibitory effect on fungal growth^42^. Due to the limitations of the available methods, we could not measure many reactive species. The concentrations of nitric oxide (NO) and hydrogen peroxide (H_2_O_2_) were measured. The levels of both reactive species increased more dramatically with compound concentration in **1** and **2** than in **3** at day 0 (Fig. 9). After 4 days, NO concentration was generally elevated in all treatments without a significant change in trend among Co-CPs (Fig. 9). However, a similar level of H_2_O_2_ (approximately 12 µM) was observed in all treatment concentrations of **2**, while the H_2_O_2_ level was decreased in the treatment with higher concentrations of **1** and **3** (Fig. 9). Our data demonstrate that Co-CPs can generate NO and H_2_O_2_, and these species may play a role in inactivating fungal cells. Recently, it is reported that ROS can be generated by photocatalysis of MOFs^43,44^. Photoinduced metal ion activates O_2_, producing ROS such as superoxide or singlet oxygen via electron transfer. Since our experiments were performed in the presence of light, light-induced electron transfer from Co metal to O_2_ might produce ROS such as superoxide or singlet oxygen. H_2_O_2_ and NO can be further generated through reaction with dissolved oxygen and nitrogen in PBS background solution. However, the relatively lower levels of NO and H_2_O_2_ observed in the treatment with **3** are not sufficient to explain the high antifungal activity of **3**. Moreover, the H_2_O_2_ level was maintained at approximately 12 µM in all treatment concentrations of **2** and reduced with the increased concentration of **1** and **3**. These results are not well matched with the antifungal effects. In addition to reactive species, other factors, such as mechanical damage to fungal cells by contact with the surface of Co-CPs or toxicity of glutarate (ligand chemical in Co-CPs) and Co^II^ ion, should also be considered.

Additionally, degradation tests were carried out in the 1x PBS solution used for antifungal testing at room temperature to confirm that the leaching of Co^II^ ions from Co-CPs was not significant. The quantities of Co^II^ ions released from Co-CPs were monitored by ICP-MS (ICP-MS measurements were performed in Seoul Center, Korea Basic Science Institute) after 1 day, 2 days, 3 days and 4 days (Fig. S13). The concentration of released Co^II^ ions from 1 mg of a compound in 1 mL of 1x PBS after 4 days was approximately 0.013 mg mL^−1^ for all three Co-CPs. The amount of Co^II^ ions released from **3** was calculated to be 0.0033 mg mL^−1^, which is negligible compared to 0.25 mg mL^−1^ of the 99.93% inactivating property. Co-CPs cannot easily release Co^II^ ions and are stable in 1x PBS. From these results, we infer that reactive species from Co-CPs, the small amount of leached Co^II^ ions, and Co-CPs themselves actively participate in fungal inactivation.

To find the toxicity of glutarate and Co^II^ ion, we incubated *C. albicans* cells and *A. niger* spores in PBS solutions containing glutarate (2 mg/ml, equivalent to Co-CPs concentration showing the highest antifungal activity) and Co salt (0.02 mg/ml, equivalent to the released Co^II^ concentration after 4 days) for 4 days and measured the fungal viability. Glutarate did not affect significantly the viability of both fungal cells (Fig. S14). However, treatment with Co salt reduced the viability of *C. albicans* cells up to around 50% while no significant effect on *A. niger* spores was observed (Fig. S14). Although reduction in *C. albicans* cell viability by Co-CPs (over 90%) was much higher than that by Co salt (around 50%), Co^II^ ion released from Co-CPs could partially contribute to fungal inactivation, probably through the redox activity of Co^II^ ion on biomolecules leading to the fungal cell damage.

In summary, three new Co-CPs containing glutarates and bipyridyl ligands, formulated as [Co_2_(Glu)_2_(µ-bpa)_2_]·(H_2_O)_4_ (**1**), [Co_4_(Glu)_4_(µ-bpp)_2_] (**2**), and [Co_2_(Glu)_2_(µ-bpe)_2_]·(H_2_O)_0.5_ (**3**), were prepared and characterized by X-ray structure determination. Glutarates bridge Co^II^ ions to form 2D sheets, and the sheets were also bridged either by bpa or by bpp ligands to form 3D networks **1** and **2**, respectively. Both **1** and **2** are two-fold interpenetrated, and there is no significant void volume in both networks. Four glutarates bridge two Co^II^ ions to form chains, and these chains are connected by bpe ligands to form the 2D sheet **3**. The antifungal properties of these new Co-CPs were tested against two model fungal pathogens, *C. albicans* and *A. niger*. Under the maximum concentration of Co-CPs, 2.0 mg mL^−1^, the inhibition rates of Co-CPs against *A. niger* were much lower (44–62%) than those (90–99.98%) observed in *C. albicans*. The results indicate that Co-CPs can inactivate *C. albicans* cells more efficiently than *A. niger* spores in the same treatment time, and the greater inactivation of *C. albicans* by Co-CPs may result from dramatic changes in the morphology of *C. albicans*. Co-CPs can generate NO and H_2_O_2_, and these species may play a role in inactivating fungal cells. Additionally, degradation tests showed that the leaching of Co^II^ ions from Co-CPs was not significant. These results imply that the small amount of leached Co^II^ ions and Co-CPs themselves as well as reactive species generated by Co-CPs can participate in fungal inactivation. Further investigation into the antifungal mechanisms of Co-CPs and other MOFs will be conducted to obtain a clear conclusion.

## Experimental Section

### Preparation of [Co_2_(Glu)_2_(µ-bpa)_2_]·(H_2_O)_4_ (1)

A mixture of Co(NO_3_)_2_·6H_2_O (0.116 g, 0.4 mmol), glutaric acid (0.053 g, 0.4 mmol), and 1,2-bis(4-pyridyl)ethane (0.147 g, 0.8 mmol) in 10 mL of *N*,*N*-dimethylformamide (DMF) was placed in a Teflon-lined high pressure vessel. The vessel was placed in an oven at 100 °C for 72 h. After cooling to room temperature, the plum purple crystalline rods were retrieved by filtration, washed with DMF, and dried in air overnight. The yield was 0.080 g. IR (KBr): ν(cm^−1^) = 3067(w), 2966(w), 2904(w), 1613(s), 1581(s), 1541(s), 1413(s), 1390(s), 1301(s), 1273(m), 1223(m), 1145(w), 1072(w), 1044(w), 1022(m), 838(s), 765(w) (Fig. S15). Anal. Calcd. for [Co_2_(C_5_H_6_O_4_)_2_(C_12_H_12_N_2_)_2_]·(H_2_O)_4_ (818.60), **1**: C, 49.89; H, 5.42; N, 6.84%. Found; C, 49.85; H, 4.71; N, 7.28%.

### Preparation of [Co_4_(Glu)_4_(µ-bpp)_2_] (2)

A mixture of Co(NO_3_)_2_·6H_2_O (0.116 g, 0.4 mmol), glutaric acid (0.053 g, 0.4 mmol), and 1,2-bis(4-pyridyl)propane (0.159 g, 0.8 mmol) in 10 mL of DMF was placed in a Teflon-lined high pressure vessel. The vessel was placed in an oven at 100 °C for 72 h. After cooling to room temperature, the purple crystalline rods were retrieved by filtration, washed with DMF, and dried in air overnight. The yield was 0.060 g. IR (KBr): ν(cm^−1^) = 3072(w), 2943(w), 2865(w), 1602(s), 1541(s), 1407(s), 1312(m), 1273(m), 1223(m), 1161(w), 1072(m), 1027(m), 1022(m), 882(w), 815(m) (Fig. S15). Anal. Calcd. for [Co_4_(C_5_H_6_O_4_)_4_(C_13_H_14_N_2_)_2_] (1152.66), **3**: C, 47.93; H, 4.55; N, 4.86%. Found; C, 47.41; H, 4.59; N, 5.15%.

### Preparation of [Co_2_(Glu)_2_(µ-bpe)_2_]·(H_2_O)_0.5_ (3)

A mixture of Co(NO_3_)_2_·6H_2_O (0.116 g, 0.4 mmol), glutaric acid (0.053 g, 0.4 mmol), and 1,2-bis(4-pyridyl)ethylene (0.146 g, 0.8 mmol) in 10 mL of DMF and distilled water (7:3 vol/vol%) was placed in a Teflon-lined high pressure vessel. The vessel was placed in an oven at 100 °C for 72 h. After cooling to room temperature, the burgundy crystalline rods were retrieved by filtration, washed with DMF, and dried in air overnight. The yield was 0.062 g. IR (KBr): ν(cm^−1^) = 3066(w), 3021(w), 2971(w), 2949(w), 2927(w), 2888(w), 1592(s), 1564(s), 1418(s), 1351(m), 1312(m), 1206(m), 1156(m), 1056(w), 1022(m), 988(m), 882(m), 838(s), 798(m), 737(w), 664(m) (Fig. S15). Anal. Calcd. for [Co_2_(C_5_H_6_O_4_)_2_(C_12_H_10_N_2_)_2_]·(H_2_O)_0.5_ (751.51), **3**: C, 54.34; H, 4.43; N, 7.46%. Found; C, 54.17; H, 4.26; N, 7.36%.

### X-ray crystallography

The X-ray diffraction data for all three Co-CPs were collected on a Bruker APX-II diffractometer equipped with a monochromator in the Mo Kα (λ = 0.71073 Å) incident beam. Each crystal was mounted on a glass fibre. The CCD data were integrated and scaled using the Bruker-SAINT software package, and the structure was solved and refined using SHEXL-2014^45^. All hydrogen atoms were placed in the calculated positions. The crystallographic data for the three compounds are listed in Table 1. The selected bond distances and angles of compounds are listed in Table 2. Structural information was deposited at the Cambridge Crystallographic Data Centre. The CCDC reference numbers are 1561941 for **1**, 1561902 for **2**, and 1561903 for **3**.

### Instrumentation

Thermogravimetric analysis was carried out on a TGA Q5000 (TA Instruments) under a nitrogen atmosphere. The elemental compositions of Co-CP samples were analysed at the Organic Chemistry Research Center, Sogang University (Seoul, Korea) by using EA1112 (CE Instruments, Italy). Powder X-ray diffraction patterns were recorded on a Bruker D8 Focus diffractometer (40 kV, 30 mA, step size = 0.02°). IR spectra were measured on a BIO RAD FTS 135 spectrometer as KBr pellets.

### Antifungal assays

We treated *C. albicans* (KCTC7270) cells and *A. niger* (130708) spores with 3 types of Co-CPs to assess the antifungal activity of Co-CPs. Both *C. albicans* and *A. niger* were maintained and propagated in PDA medium. To obtain *C. albicans* cells for the assay, 1–2 colonies from PDA plates were suspended in 1 mL of PDB (potato dextrose broth). Then, 10 µL of suspension was inoculated into 15 mL of PDB placed in a 50 ml conical tube. The tube was incubated at 30 °C with shaking for 16–20 h and then centrifuged at 3,134 × *g* for 5 min. After the liquid part was discarded, the *C. albicans* cell pellet was washed with 1x PBS. The washed pellet was resuspended in new PBS, and the cell concentration was adjusted to 10^8^ per mL.

Spores of *A. niger* were harvested from 2- to 3-week-old PDA culture plates. Approximately 20 mL of sterile deionized (DI) water was added to each PDA culture plate, and fungal materials were scraped using a plastic scraper. The resulting suspension was filtered through 3 layers of sterile Miracloth (Millipore, Burlington, MA, USA) to remove fungal mycelia. The spore suspension was centrifuged at 3,134 × *g* for 5 min, and the liquid was discarded. The spore pellet was washed with 1x PBS and then resuspended in fresh 1x PBS, and the spore concentration was adjusted to 10^8^ per ml.

For treatment with Co-CPs, a suspension of *C. albicans* cells or *A. niger* spores was placed in a 24-well plate (1 mL per well). Then, the Co-CP (suspended in PBS) was added to each well to the indicated concentrations (2, 1, 0.5, 0.25, 0.125 mg mL^−1^). The well plate was shaken at room temperature for 4 days. The treated suspension was serially diluted, and then 100 µL of diluted suspension was spread on PDA plates. The plates were incubated at 30 °C for 2 days, and the number of colonies was counted. Three replicates of treatment were performed at each compound concentration, and colonies were counted from 9 replicate plates.

### Analysis of morphology by scanning electron microscopy (SEM)

Morphological changes in fungal cells and spores were analysed using SEM following Co-CP treatment. After treatment with Co-CPs (2 mg mL^−1^) for 4 days, control (no Co-CPs) and treated *C. albicans* cells and *A. niger* spores were washed twice with PBS and fixed in Karnovsky’s fixative [2% (v/v) paraformaldehyde and 2% (v/v) glutaraldehyde in 1x PBS] overnight at 4 °C. After primary fixation, the fungal cells and spores were washed three times with PBS. The washed cells and spores were mixed with approximately 1 ml of 1% (v/v) osmium tetroxide (Ted Pella Inc., Redding, CA, USA) and incubated for 2 h at room temperature in the dark. The cells and spores were then washed twice with PBS and dehydrated via serial incubations in 30, 50, 70, 80, 90, and 100% (twice) ethanol. In each dehydration step, the cells and spores were mixed with 1 ml of ethanol and incubated for 5 min at 4 °C. After dehydration, 500 µL of hexamethyldisilazane (HMDS) was applied to the samples, and the samples were incubated for 15 min for drying. This drying step was repeated once. Then, the dried cells and spores were mounted on carbon tape, coated with platinum, and examined under a scanning electron microscope (SEM) (JEOL, Tokyo, Japan).

### Measurement of NO (nitric oxide) and H_2_O_2_ concentration

To analyse NO and H_2_O_2_ concentrations in 1x PBS solution treated with Co-CPs, 1 mL of PBS solution was placed in each well of a plate, and the Co-CPs (suspended in PBS) were added at the indicated concentrations (2, 1, 0.5, 0.25, 0.125 mg mL^−1^). The well plate was shaken at room temperature for 4 days. The concentrations of NO and H_2_O_2_ were measured immediately after Co-CPs were added (0 day) and after 4 days. The NO and H_2_O_2_ levels were measured using a QuantiChromTM Nitric Oxide Assay Kit (BioAssay Systems, Hayward, CA, USA) and an Amplex™ Red Hydrogen Peroxide/Peroxidase Assay Kit (Molecular Probes, Eugene, OR, USA), respectively, following the manufacturer’s protocols.

### Co-CP degradation and release of Co^II^ ion

To assess whether Co-CPs will be able to release their constituent Co^II^ ions, 1 mL of 1x PBS solution was added to 1 mg of each compound and stirred for 1 day–4 days at r.t. After different times, each sample was centrifuged, and the supernatant was removed from the reaction tube. The amount of Co^II^ released in the sample was quantified by ICP-MS (ICP-MS measurements were performed in Seoul Center, Korea Basic Science Institute). The degree of degradation is represented as the concentration of Co^II^ ions released into the medium.

## Supplementary information

Supplementary information

## References

[1] Yaghi OM (2003). Reticular synthesis and the design of new materials. Nature.

[2] Kim J (2001). Assembly of metal-organic frameworks from large organic and inorganic secondary building units:  new examples and simplifying principles for complex structures. J. Am. Chem. Soc..

[3] Foo ML, Matsuda R, Kitagawa S (2014). Functional hybrid porous coordination polymers. Chem. Mater..

[4] Eddaoudi M (2015). Zeolite-like metal–organic frameworks (ZMOFs): design, synthesis, and properties. Chem. Soc. Rev..

[5] He Y, Zhou W, Krishnad R, Chen B (2012). Microporous metal–organic frameworks for storage and separation of small hydrocarbons. Chem. Commun..

[6] Cadiau A, Adil K, Bhatt PM, Belmabkhout Y, Eddaoudi M (2016). A metal-organic framework*–*based splitter for separating propylene from propane. Science.

[7] Cui X (2016). Pore chemistry and size control in hybrid porous materials for acetylene capture from ethylene. Science.

[8] Li J-R (2011). Carbon dioxide capture-related gas adsorption and separation in metal-organic frameworks. Coord. Chem. Rev..

[9] Sumida K (2012). Carbon dioxide capture in metal-organic frameworks. Chem. Rev..

[10] Bae Y-S, Snurr RQ (2011). Development and evaluation of porous materials for carbon dioxide separation and capture. Angew. Chem., Int. Ed..

[11] Eberle U, Felderhoff M, Schüth F (2009). Chemical and physical solutions for hydrogen storage. Angew. Chem., Int. Ed..

[12] Seayad AM, Antonelli DM (2004). Recent advances in hydrogen storage in metal-containing inorganic nanostructures and related materials. Adv. Mater..

[13] Suh MP, Park HJ, Prasad TK, Lim D-W (2012). Hydrogen storage in metal-organic frameworks. Chem. Rev..

[14] Farrusseng D, Aguado S, Pinel C (2009). Metal-organic frameworks: opportunities for catalysis. Angew. Chem., Int. Ed..

[15] Dhakshinamoorthy A, Opanasenko M, Čejka J, Garcia H (2013). Metal organic frameworks as heterogeneous catalysts for the production of fine chemicals. Catal. Sci. Technol..

[16] Gu J-M, Kim W-S, Huh S (2011). Size-dependent catalysis by DABCO-functionalized Zn-MOF with one-dimensional channels. Dalton Trans..

[17] Horcajada P (2012). Metal-organic frameworks in biomedicine. Chem. Rev..

[18] Cunha D (2013). Rationale of drug encapsulation and release from biocompatible porous metal−organic frameworks. Chem. Mater..

[19] Hwang IH (2012). Bifunctional 3D Cu-MOFs containing glutarates and bipyridyl ligands: selective CO_2_ sorption and heterogeneous catalysis. Dalton Trans..

[20] Hwang IH (2013). Zn-MOFs containing flexible α,ω-alkane (or alkene)-dicarboxylates and 1,2-bis(4-pyridyl)ethane ligands: CO_2_ sorption and photoluminescence. Cryst. Growth Des..

[21] Kim, H.-C. *et al*. Zn-MOFs containing flexible α,ω-alkane (or alkene)-dicarboxylates with 1,2-bis(4-pyridyl)ethylene: comparison with Zn-MOFs containing 1,2-bis(4-pyridyl)ethane ligands. *Cryst. Eng. Comm*, **19**, 99–109 (2017) and references there in.

[22] Bezuidenhout CX, Smith VJ, Bhatt PM, Esterhuysen C, Barbour LJ (2015). Extreme carbon dioxide sorption hysteresis in open-channel rigid metal–organic frameworks. Angew. Chem. Int. Ed..

[23] Bezuidenhout CX, Smith VJ, Esterhuysen C, Barbour LJ (2017). Solvent- and pressure-induced phase changes in two 3D copper glutarate-based metal–organic frameworks via glutarate (+gauche ⇄ −gauche) conformational Isomerism. J. Am. Chem. Soc..

[24] Rather, B. & Zaworotko, M. J. A 3D metal-organic network, [Cu_2_(glutarate)_2_(4,4’-bipyridine)], that exhibits single-crystal to single-crystal dehydration and rehydration, *Chem. Commun*. 830–831 (2003). 10.1039/b301219k 12739633

[25] Chen B (2008). Metal-organic framework with rationally tuned micropores for selective adsorption of water over methanol. Inorg. Chem..

[26] Seco JM (2013). Modular structure of a robust microporous MOF based on Cu_2_ paddle-wheels with high CO_2_ selectivity. Chem. Commun..

[27] Jo JH, Kim H-C, Huh S, Kim Y, Lee DN (2019). Antibacterial activities of Cu-MOFs containing glutarates and bipyridyl ligands. Dalton Trans..

[28] Kabir MA, Hussain MA, Ahmad Z (2012). *Candida albicans*: a model organism for studying fungal pathogens. ISRN Microbiol..

[29] Baker SE (2006). *Aspergillus niger* genomics: past, present and into the future. Med. Mycol..

[30] Bouson S, Krittayavathananon A, Phattharasupakun N, Siwayaprahm P, Sawangphruk M (2017). Antifungal activity of water**-**stable copper**-**containing metal**-**organic frameworks. R. Soc. open sci..

[31] Chiericatti C, Basillico JC, Zapata ML, Zamaro JM (2012). Novel application of HKUST-1 metal-organic framework as antifungal: Biologicla tests and physicochemical characterizations. Micropor. Mesopor. Mater..

[32] Zhuang W (2012). Highly potent bactericidal activity of porous metal-organic frameworks. Adv. Healthcare Mater..

[33] Spek, A. L. *PLATON-A multipurpose Crystallographic Tool*, Utrecht University, Utrecht, The Netherlands, (2004).

[34] Blatov VA, Carlucci L, Ciani G, Proserpio DM (2004). Interpenetrating metal–organic and inorganic 3D networks: a computer-aided systematic investigation. Part I. Analysis of the Cambridge structural database. CrystEngComm.

[35] Jaros SW (2016). Silver(I) 1,3,5-Triaza-7-phosphaadamantane Coordination Polymers Driven by Substituted Glutarate and Malonate Building Blocks: Self-Assembly Synthesis, Structural Features, and Antimicrobial Properties. Inorg. Chem..

[36] Jaros SW (2016). Bioactive Silver−Organic Networks Assembled from 1,3,5-Triaza-7-phosphaadamantane and Flexible Cyclohexanecarboxylate Blocks. Inorg. Chem..

[37] Jaros SW (2014). Aliphatic Dicarboxylate Directed Assembly of Silver(I) 1,3,5-Triaza-7-phosphaadamantane Coordination Networks: Topological Versatility and Antimicrobial Activity. Cryst. Growth Des..

[38] Jaros SW (2013). New silver BioMOFs driven by 1,3,5-triaza-7-phosphaadamantane-7-sulfide (PTALS): synthesis, topological analysis and antimicrobial activity. CrystEngComm.

[39] Lara HH (2015). Effect of silver nanoparticles on *Candida albicans* biofilms: an ultrastructural study. J. Nanobiotechnol.

[40] Dutta RK, Nenavathu BP, Gangishetty MK, Reddy AV (2012). Studies on antibacterial activity of ZnO nanoparticles by ROS incuced lipid peroxidation. Colloid. Surface. B: Biointerface..

[41] Sawangphruk M, Srimuk P, Chiochan P, Sangsri T, Siwayaprahm P (2012). Synthesis and antifungal activity of reduced graphene oxide nanosheets. Carbon.

[42] Fu PP, Xia Q, Hwang H-M, Ray PC, Yu H (2014). Mechanisms of nanotoxicity: generation of reactive oxygen species. J. Food Drug Anal..

[43] Liu Y, Howarth AJ, Hupp JT, Farha OK (2015). Selective photooxidation of a mustard-gas simulant catalyzed by a porphyrinic metal-organic framework. Angew. Chem. Int. Ed..

[44] Li P (2019). Metal-organic frameworks with photocatalytic bactericidal activity for integrated air cleaning. Nat. Comm..

[45] Sheldrick G (2015). Crystal structure refinement with SHELXL. Acta Crystallogr. Sect. C: Struct. Chem..

