# Supplementary material for: Cobalt(II)-coordination polymers containing glutarates and bipyridyl ligands and their antifungal potential
Source: Sci Rep. 2019 Oct 18;9:14983. doi: 10.1038/s41598-019-50258-1 (PMC6800428; doi:10.1038/s41598-019-50258-1)
Supplement: Supplementary file 1 — Supplementary information [file 41598_2019_50258_MOESM1_ESM.docx]

***Supporting Information***

**Cobalt(II)-coordination polymers containing glutarates and bipyridyl ligands and their antifungal potential**

Hyun-Chul Kim,^1,†^ Sarmistha Mitra,^2,†^ Mayura Veerana,^2,†^ Jun-Sup Lim,^2^ Hye-Ryeon Jeong,^2^ Gyungsoon Park,^*,2^ Seong Huh,^1^ Sung-Jin Kim,^3^ and Youngmee Kim^*,3^

^1^ Department of Chemistry and Protein Research Centre for Bio-Industry, Hankuk University of Foreign Studies, Yongin 449-791, Korea.

^2^ Plasma Bioscience Research Center and Department of Electrical and Biological Physics, Kwangwoon University, Seoul 01897, Republic of Korea. E-mail: [gyungp@kw.ac.kr](mailto:gyungp@kw.ac.kr)

*^3^ Institute of Nano-Bio Technology and Department of Chemistry and Nano Science, Ewha Womans University, Seoul 120-750, Korea. E-mail:* [*ymeekim@ewha.ac.kr*](mailto:ymeekim@ewha.ac.kr)*; Fax: +82-2-3277-2384; Tel: +82-2-3277-3589*

^†^ These authors contributed equally to this work.

Correspondence and requests for materials should be addressed to Y.K. (email: [ymeekim@ewha.ac.kr](mailto:ymeekim@ewha.ac.kr)) or to G.P. (email: [gyungp@kw.ac.kr](mailto:gyungp@kw.ac.kr)).


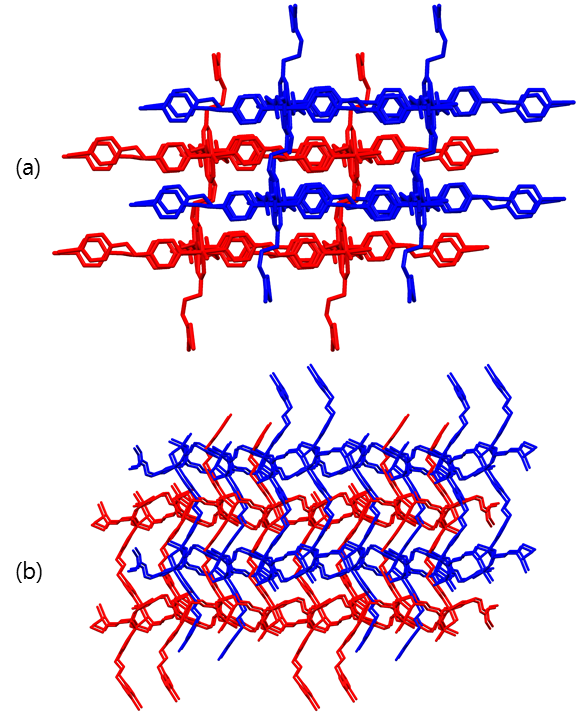


**Figure S1**. Interpenetrated 3D networks of **1** (a) and **2** (b) are shown in different colours. All hydrogens and disordered atoms are omitted for clarity.


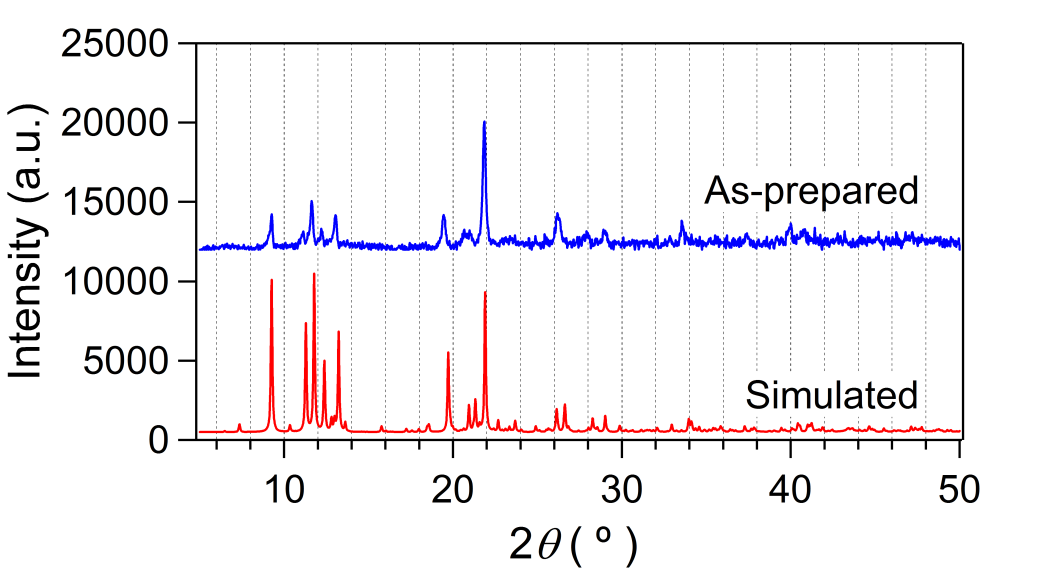


**Figure S2.** PXRD patterns of **1**.


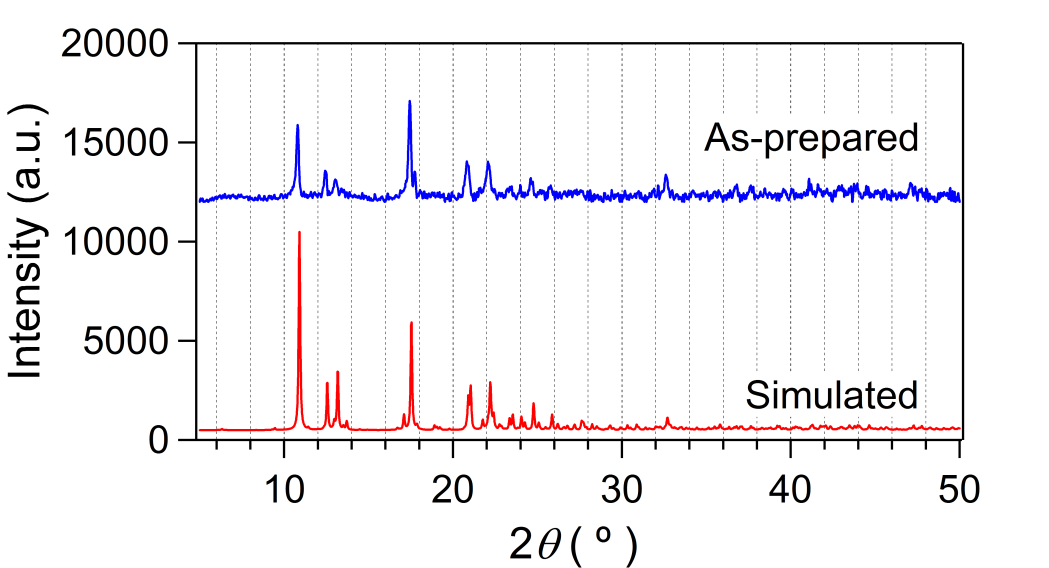


**Figure S3.** PXRD patterns of **2**.


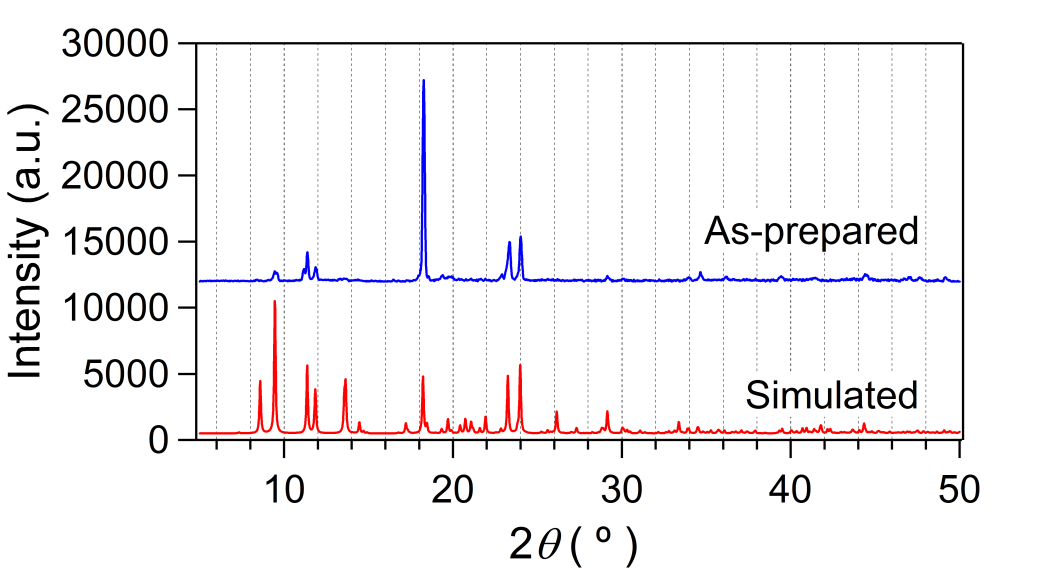


**Figure S4.** PXRD patterns of **3**.


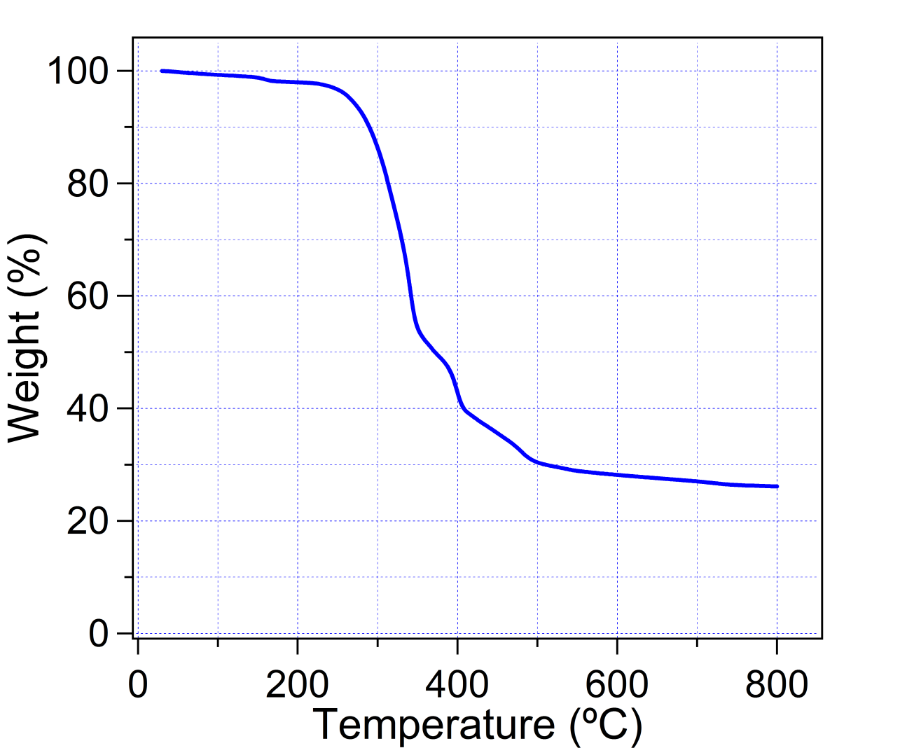


8.8% 4H_2_O

**Figure S5.** TGA profile for **1**.


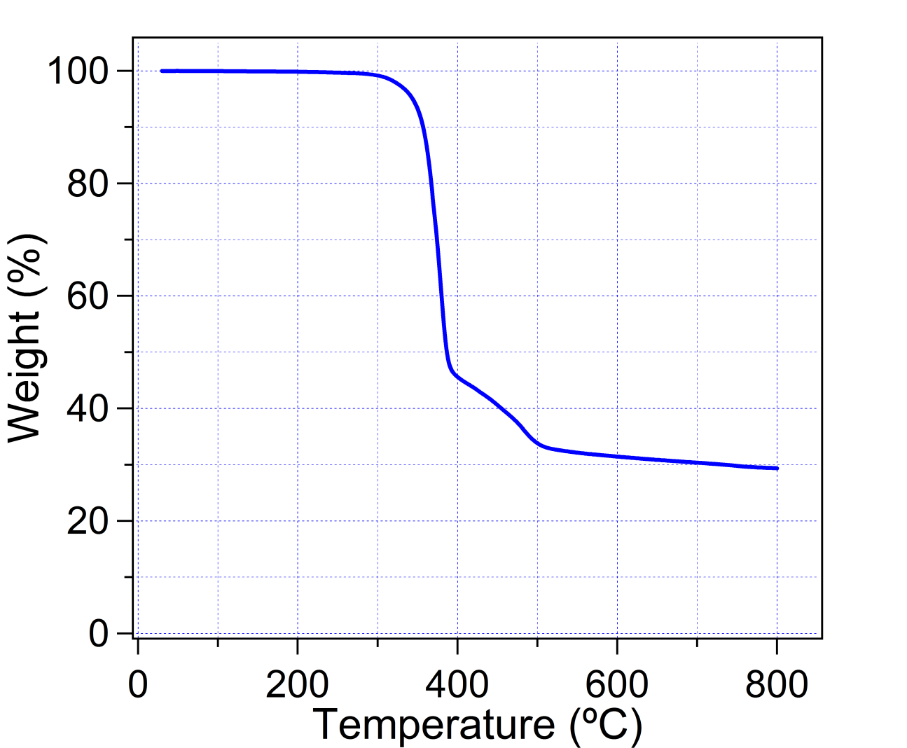


**Figure S6.** TGA profile for **2**.


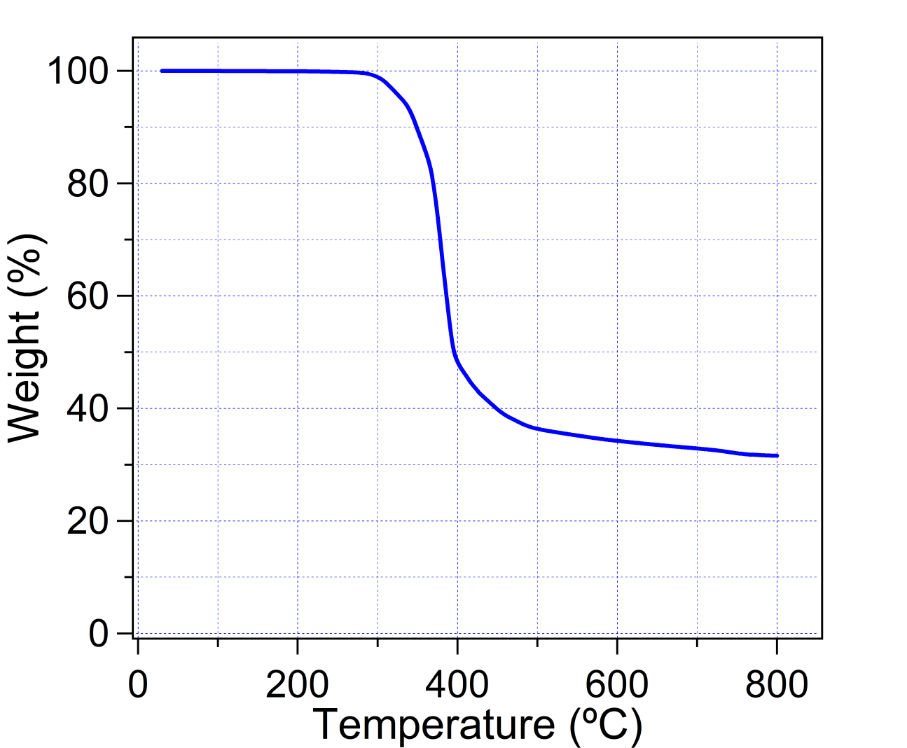


**Figure S7.** TGA profile for **3**.


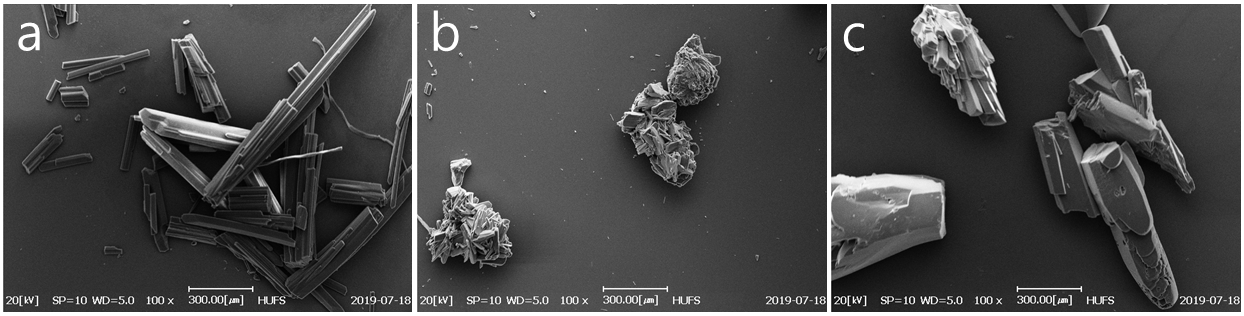


**Figure S8.** SEM images of **1** (a), **2** (b), and **3** (c) with the same magnification (x100). Scale bars = 300 μm.


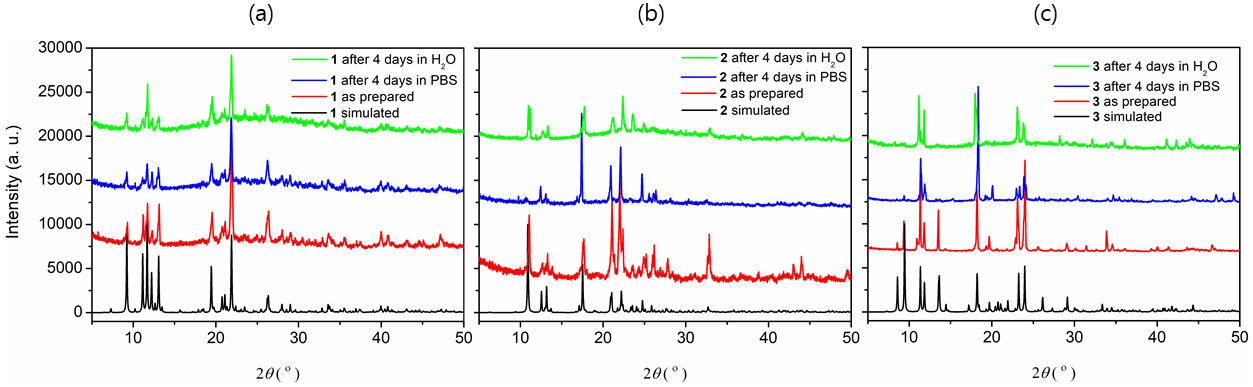


**Figure S9.** PXRD patterns of **1** (a), **2** (b), and **3** (c) after 4 days in water and 1x PBS solution.


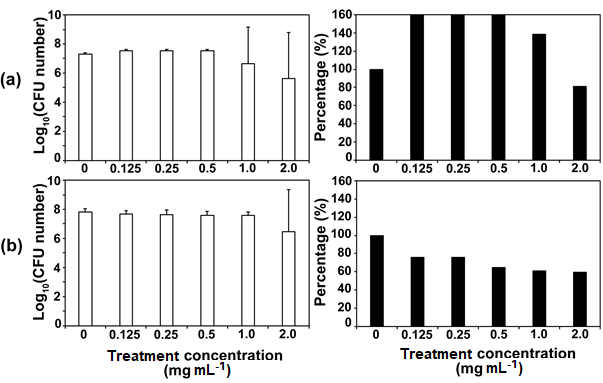


**Figure S10**. Antifungal activities of **3** against *C. albicans* (a) and **1** against *A. niger* (b) in different concentrations after 1-day incubation. Log scale CFU (colony forming unit) numbers of *C. albicans* in each treatment (left graph). Percentage calculated as follows; (CFU number in each compound treatment / CFU number of control) x 100 (right graph). Each bar represents averages and standard diviations of 9 replicates.

**
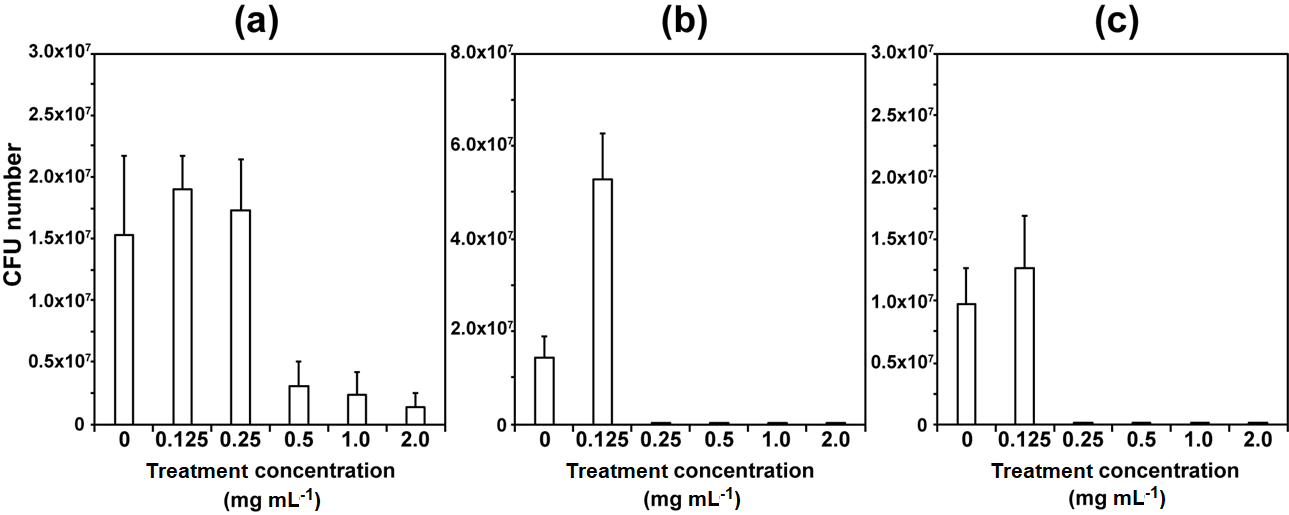
**

**Figure S11.** Average CFU (colony forming unit) numbers of *C. albicans* for **1** (a), **2** (b), and **3** (c). Each bar represents averages and standard diviations of 9 replicates.


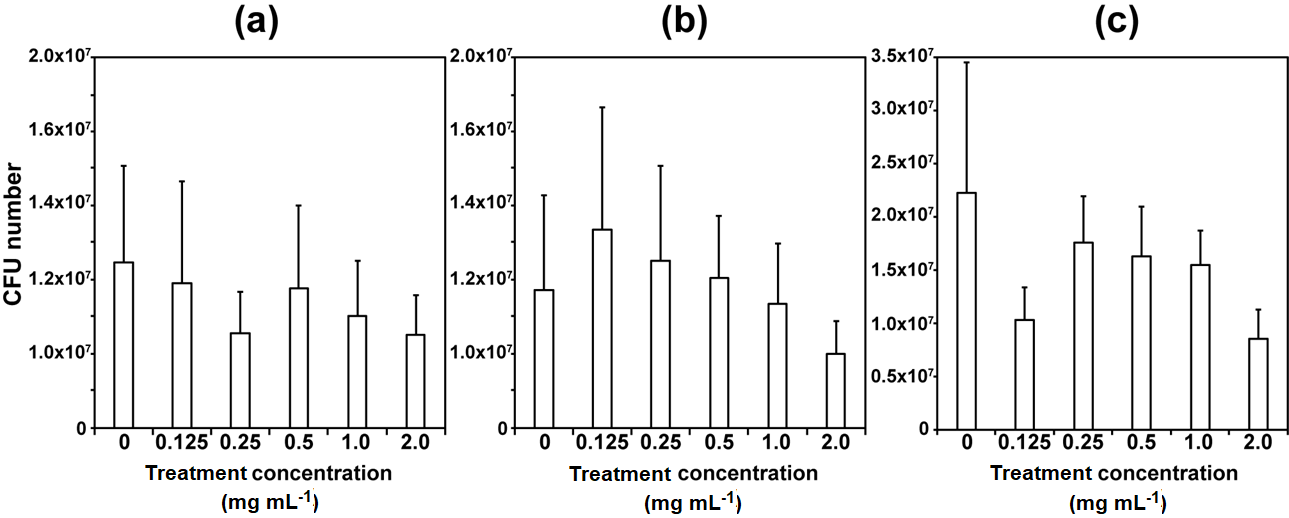


**Figure S12.** Average CFU (colony forming unit) numbers of *A. niger* for **1** (a), **2** (b), and **3** (c). Each bar represents averages and standard diviations of 9 replicates.


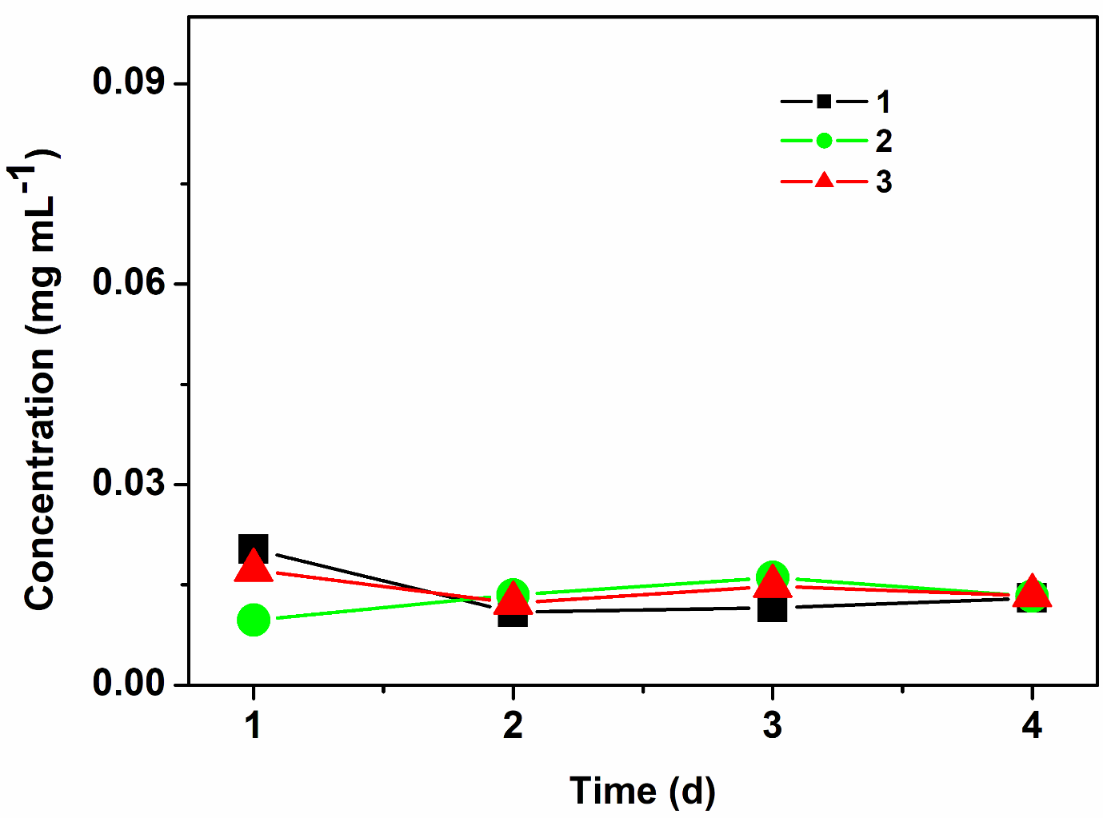


**Figure S13.** Concentrations of Co^II^ released from 1 mg of Co-CPs in 1 mL of 1x PBS solution after 1 day, 2 days, 3 days, and 4 days.


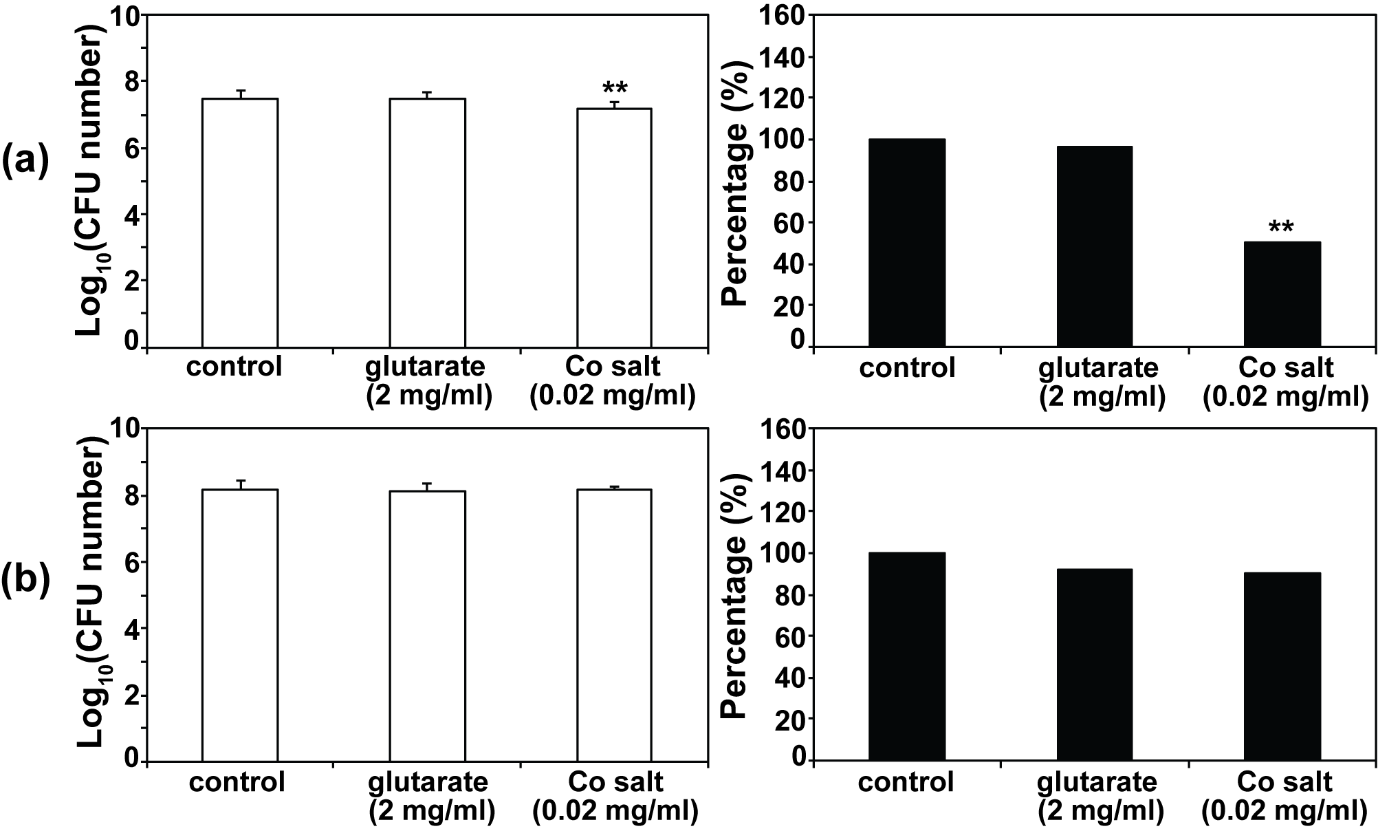


**Figure S14.** Antifungal effects of glutarate and Co^II^ in 1x PBS solution against *C. albicans* (a) and *A. niger* (b) after 4 days. Each value is the average of 9 replicates. **, *p*<0.01


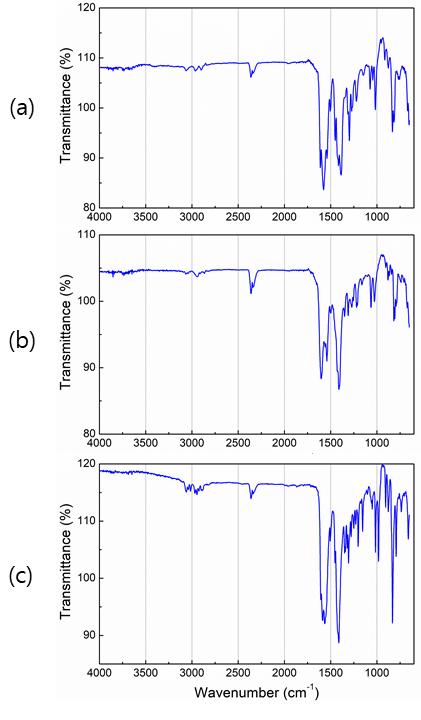


**Figure S15.** IR spectra of **1** (a), **2** (b) and **3**(c).
